# A child with household transmitted COVID-19

**DOI:** 10.1186/s12879-020-05056-w

**Published:** 2020-05-07

**Authors:** Li-juan Mao, Jian Xu, Zhi-hao Xu, Xiao-ping Xia, Bin Li, Jian-guo He, Peng Zhao, Jian-wei Pan, Dan Zhang, Yue Su, Yue-hong Wang, Zhe-feng Yuan

**Affiliations:** 1 Department of Pediatrics, The Fourth Affiliated Hospital of Zhejiang University School of Medicine, N1 Shangcheng Avenue, Yiwu, 322000 Zhejiang Province China; 2 Department of Obstetrics and Gynecology, The Fourth Affiliated Hospital of Zhejiang University School of Medicine, Yiwu, China; 3 Department of Respiratory and Critical Care Medicine, The Fourth Affiliated Hospital of Zhejiang University School of Medicine, Yiwu, China; 4 Department of Clinical Laboratory, The Fourth Affiliated Hospital of Zhejiang University School of Medicine, Yiwu, China; 5 Department of Infectious Diseases, The Fourth Affiliated Hospital of Zhejiang University School of Medicine, Yiwu, China; 6 Department of Otolaryngology, The Fourth Affiliated Hospital of Zhejiang University School of Medicine, Yiwu, China

**Keywords:** COVID-19, SARS-CoV-2, Children

## Abstract

**Background:**

Although people of all ages are susceptible to the novel coronavirus infection, which is presently named “Coronavirus Disease 2019” (COVID-19), there has been relatively few cases reported among children. Therefore, it is necessary to understand the clinical characteristics of COVID-19 in children and the differences from adults.

**Case presentation:**

We report one pediatric case of COVID-19. A 14-month-old boy was admitted to the hospital with a symptom of fever, and was diagnosed with a mild form of COVID-19. The child’s mother and grandmother also tested positive for SARS-CoV-2 RNA. However, the lymphocyte counts were normal. The chest computed tomography (CT) revealed scattered ground glass opacities in the right lower lobe close to the pleura and resorption after the treatment. The patient continued to test positive for SARS-CoV-2 RNA in the nasopharyngeal swabs and stool at 17 days after the disappearance of symptoms.

**Conclusion:**

The present pediatric case of COVID-19 was acquired through household transmission, and the symptoms were mild. Lymphocyte counts did not significantly decrease. The RNA of SARS-CoV-2 in stool and nasopharyngeal swabs remained positive for an extended period of time after the disappearance of symptoms. This suggests that attention should be given to the potential contagiousness of pediatric COVID-19 cases after clinical recovery.

## Background

Several cases of pneumonia caused by an unknown etiological agent was reported in the Chinese city of Wuhan in December 2019. The full genome sequence analysis revealed the etiological agent to be a coronavirus that is divergent from the coronaviruses that caused severe acute respiratory syndrome (SARS) and Middle East respiratory syndrome (MERS). Furthermore, all infected cases appeared to correlate to a local fresh seafood and wild animal market [1, 2]. This new coronavirus was initially named the “2019 novel coronavirus” (2019-nCoV), but was later renamed “severe acute respiratory syndrome coronavirus 2” (SARS-CoV-2) by the World Health Organization (WHO). The disease caused by SARS-CoV-2 was “Coronavirus Disease 2019” (COVID-19).

SARS-CoV-2 infection rapidly spread to all parts of China and abroad, leading to a global outbreak. According to the WHO-China joint mission report published on February 28, 2020, a cumulative total of 75,465 cases of COVID-19 have been reported in China. The median age was 51 years old (range: 2 days old to 100 years old; IQR: 39–63 years old), and merely 2.4% of these cases were under 18 years old, suggesting that cases in children are relatively uncommon [2].

It remains unclear whether children are less susceptible to the infection, or whether they show a different clinical presentation. Since COVID-19 is a novel infection and no pre-existing immunity exists in humans, everybody should theoretically have the same susceptibility to the infection. However, although 13.8% of adults develop severe disease and 6.1% of adults develop critical disease, merely 2.5% of infected children develop severe disease and 0.6% of infected children develop critical disease, suggesting that children become less ill from the infection [2]. This was also confirmed by previous case reports on COVID-19 in children [3–5]. Due to the limited availability of diagnostic tests, testing has often focused on those who are more ill, which likely resulted in the underrepresentation of milder pediatric infections at the population level.

The present case report describes the diagnosis and treatment of COVID-19 in a 14-month-old boy, with the aim of increasing our understanding of the clinical characteristics of COVID-19 in children and their differences from adults.

## Case presentation

A 14-month-old previously healthy boy sought medical care at the fever clinic of our hospital with” fever for 2 days” as the chief complaint. The child developed fever on the evening of January 31, 2020. The child’s body temperature was 38.4 °C, and had dry cough, runny nose and decreased appetite. The child had a normal birth and growth history, and received vaccinations according to plan. Furthermore, the child was breastfed and ate age-appropriate food. The child tested positive for SARS-CoV-2 by reverse transcription polymerase chain reaction (RT-PCR) in the nasopharyngeal swab in the evening of February 1, 2020, and was admitted to the hospital with symptoms of fever, dry cough and running nose. The patient’s mother also tested positive for SARS-CoV-2 by RT-PCR, and was admitted at the same time.

The physical examination at admission revealed the following: body temperature 37.6 °C (ear temperature), respiratory rate 23/min, pulse rate 105 beats/min, blood pressure 95/56 mmHg (1 mmHg = 0.133 kPa), and SPO2 99%. The child was fully conscious, and had a good response. The skin and mucosa were intact, with no signs of rash or bleeding points. The anterior fontanel was closed. The conjunctivae were clear without exudates or hemorrhage. The sclera was non-icteric. There was mild throat congestion, but no enlargement of tonsils. The patient presented no signs of respiratory distress. The breath sounds were clear bilaterally in all lobes, without rhonchi or crackles. The heart rate and rhythm were normal. No murmurs, gallops, or rubs are auscultated. The abdomen was soft and flat, with no signs of pain upon palpation. No masses, hepatomegaly, or splenomegaly were noted. The neurological examination was normal. The capillary refill was 2 seconds.

**Fig. 1:**
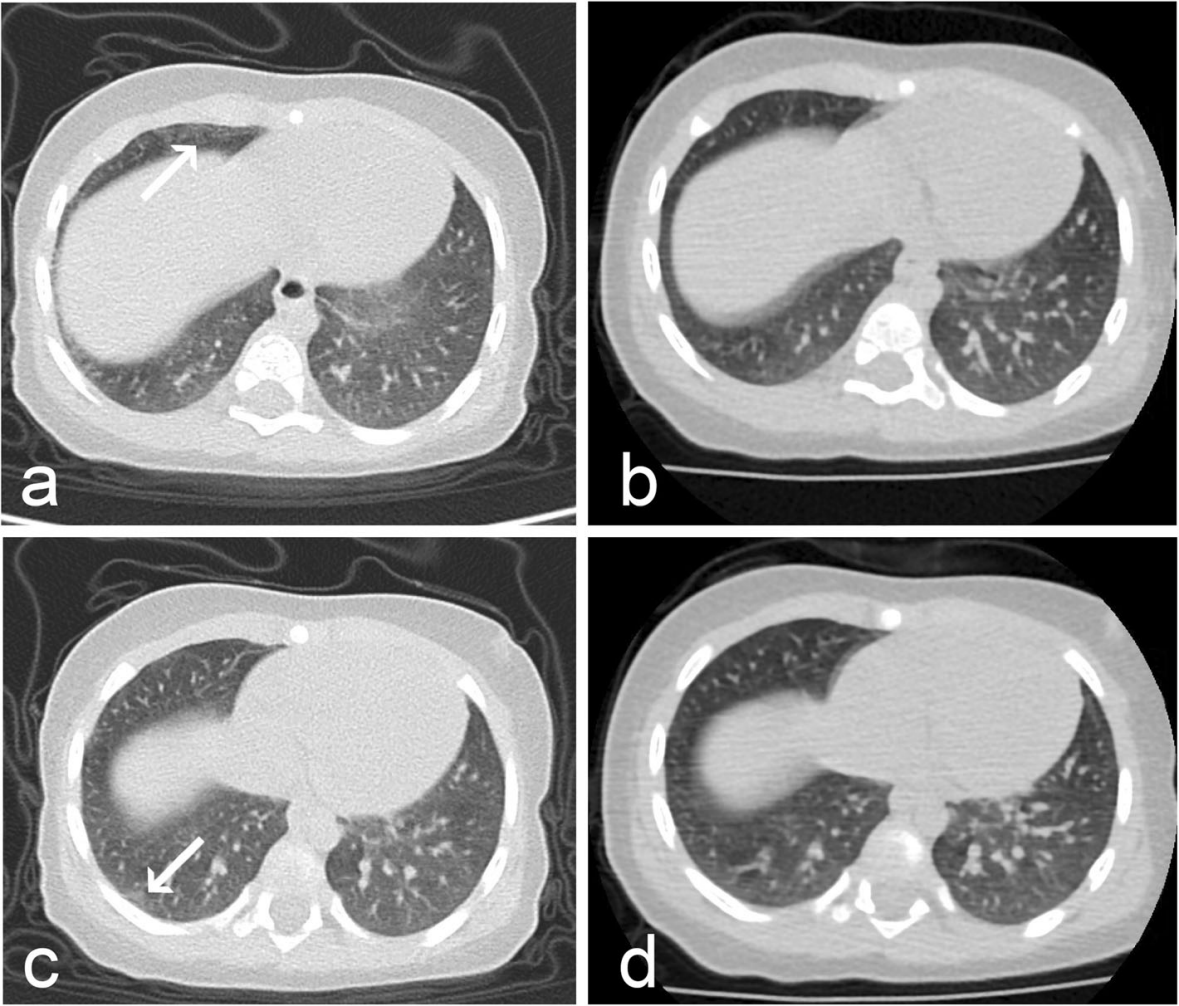
The chest CT on February 5 shows scattered ground glass opacities in the right lower lobe close to the pleura (**a**, **c**), and the reevaluation of chest CT on February 28 shows the resorption of ground glass opacities (**b**, **d**)

Laboratory examinations were performed on February 2 after admission. The results revealed the following. Nasopharyngeal swabs revealed negative results (colloidal gold-based immunochromatographic method) for influenza A and B virus, respiratory syncytial virus and adenovirus. In addition, the influenza A and B virus RNA (PCR method) was also found to be negative. Routine blood count: white blood cells 7 × 109/L (neutrophil 37.5%, lymphocyte 50.3% and monocyte 10.1%), hemoglobin 107 g/L, and platelets 353 × 109/L;C-reactive protein 7.6 mg/L; serum procalcitonin (PCT) 0.138 μg/L, serum aspartate aminotransferase 43 U/L, alanine aminotransferase 17 U/L, lactate dehydrogenase 223 U/L, creatine kinase isoenzyme 291.8 U/L, and creatinine 24 μmol/L; the coagulation analysis was normal and D-dimer 0.17 mg/L; immunoglobulin + complement: IgG 4.87 g/L, IgA 0.2 g/L, IgM 0.66 g/L, C3 0.69 g/L and C4 0.19 g/L; the T lymphocyte, B lymphocyte and NK lymphocyte subset analysis was normal; routine urine and stool analysis was normal. The chest computed tomography (CT) on February 5 revealed scattered ground glass opacities in the right lower lobe close to the pleura (Fig. 1a, c).

The possible route of transmission was investigated, and the following was revealed. The patient attended a family gathering event together with the patient’s mother and grandmother on January 20. One person who also attended the gathering recently went to Wuhan, but was asymptomatic at that time. The patient was subsequently diagnosed with COVID-19 on January 28. The child’s grandmother started to present symptoms of fever and minor cough at 3 days after the gathering. She tested positive for SARS-CoV-2 by RT-PCR in the nasopharyngeal swab (negative results in urine and stool) on January 29, and was admitted to the hospital for treatment. Her chest CT presented signs of viral pneumonia. The patient’s mother presented with a symptom of nasal congestion without fever on January 31, and tested positive for SARS-CoV-2 by RT-PCR in the nasopharyngeal swab on February 1. The virus RNA in stool, urine, vaginal discharge and breast milk were all negative despite the repeated testing. The chest CT indicated viral pneumonia. The patient’s father tested negative for SARS-CoV-2 RNA on repeated tests.

After admission, the patient was treated with 1.5 million units of recombinant human interferon α-2b by aerosolization, twice daily, during a course of 7 days, and also received other symptomatic treatment. On the second day of admission, the patient’s body temperature returned to normal. The patient continued to have occasional dry cough, but the runny nose and appetite improved. The symptoms were completely relieved on February 8, with the disappearance of fever, cough and runny nose.

The test results for SARS-CoV-2 after admission: On February 5 (at 6 days after symptom onset), the virus RNA in urine was negative. On February 6, 7, 10, 14 and 25 (at 7, 8, 11, 15 and 26 days after symptom onset), the virus RNA in the nasopharyngeal swabs were positive. During the same period, virus RNA was also found to be positive in stool. On February 27 and 29 (at 28 and 30 days after symptom onset), the virus RNA tests were repeatedly negative in both the nasopharyngeal swabs and stool. The reevaluation of the chest CT on February 28 also revealed the resorption of ground glass opacities that were previously observed in the right lower lobe (Fig. 1b, d). The patient was discharged on February 29.

## Discussion and conclusions

Coronaviruses are a large family of viruses that originate from animals, and are known to cause respiratory infections in humans. Most coronaviruses only cause mild symptoms in humans, such as HCoV-OC43, HCoV-229E, HCoV-NL63 and HCoV-HKU1. However, the coronavirus that caused the SARS outbreak (SARS-CoV) in 2003 had a fatality rate of 10% in average, depending on the infected patient’s age. This infected over 8000 individuals globally, and caused nearly 800 deaths, with the majority in China. The fatality rate for MERS, which is also caused by the coronavirus MERS-CoV, was as high as approximately 50% for those who seeked medical attention, and this claimed more than 800 lives in over 27 countries since its outbreak in 2012 [6].

The available data on the newly discovered SARS-CoV-2 suggests that it is much less fatal than the coronaviruses that caused SARS and MERS. The overall fatality rate was 3.4%, and the risk of death increases with old age. At the age over 80, the fatality rate is 14.8% [2]. Despite this relatively low fatality rate, SARS-CoV-2 has already claimed over 3100 lives globally due to its apparent ability to effectively and rapidly spread within the society and across borders. Its contagiousness appears to be much higher than SARS and MERS.

However, despite its high contagiousness, cases in children are relatively uncommon. Merely 2.4% of cases are under 18 years old. Children also appears to develop a milder disease, when compared to adults. Furthermore, 13.8% of adults develop severe disease and 6.1% of adults develop critical disease, while merely 2.5% of infected children develop severe disease and 0.6% of infected children develop critical disease [2]. A recent study that reviewed the epidemiological characteristics of 2143 children with COVID-19 found that over 90% of the cases were asymptomatic, mild, or moderate cases, and only one 14-year-old boy from Hubei province died from infection [7]. Although mortality cases in children are rare, some cases are still being brought to public attention. The Governor of Connecticut Ned Lamont confirmed the death of a COVID-19 positive 6-week-old newly born baby on his Twitter on April 1, which may be the youngest COVID-19 fatality case reported [8].

According to the WHO-China joint report, infected children were commonly identified through contact tracing in the households of infected adults, which was also the case in this study [2]. Other case reports of COVID-19 in children all support household or family cluster transmission [3–5]. Therefore, the transmission route for SARS-CoV-2 in children appears to be similar to that of SARS-CoV and MERS-CoV [9, 10].

The patient tested positive for SARS-CoV-2 RNA in both the nasopharyngeal swabs and stool long after the resolution of clinical symptoms. Although the clinical symptoms already resolved at 9 days after symptom onset, the virus RNA in the nasopharyngeal swabs and stool first turned negative at 28 days after symptom onset. This suggests that the contagiousness of the patient remains unclear, despite clinical recovery. Persistently positive nucleic acid tests have also been reported in adults. A recent letter to the Journal of American Medical Association (JAMA) reported four patients with COVID-19, who met the criteria for hospital discharge or the discontinuation of quarantine in China (the absence of clinical symptoms and radiological abnormalities, and two negative RT-PCR test results), and had positive RT-PCR test results after 5–13 days [11]. However, the clinical significance of positive RNA in stool as the possible transmission via the fecal-oral route remains unclear, and still requires further confirmation. Nevertheless, these results suggest the need to extend the monitoring period of patients after clinical recovery and hospital discharge, and take caution in handling the excretions of COVID-19 patients.

It remains unclear whether children are less susceptible to the infection, or whether they show a different clinical presentation. During the SARS and MERS outbreak, cases in children have been under-reported [9, 10], and this is likely also the case during the present COVID-19 outbreak. Due to the limited availability of diagnostic tests, testing has often focused on those who are more ill, which likely resulted in the underrepresentation of milder pediatric infections at the population level. A latest research suggested that children are as susceptible to the SARS-CoV-2 virus as adults. When looking into the close contact of people diagnosed with COVID-19, children under 10 years old, who were potentially exposed to the virus, were shown to be just as likely to become infected as the other age groups, with an approximately 7–8% chance of later testing positive. The same study also confirmed the role of household transmission as the major transmission route. Hence, for people who lived in the same household, someone would approximately be six times more likely to get infected, when compared to people who made contact in other settings [12].

It has been suggested that children might show less severe symptoms of COVID-19 due to the immaturity of viral receptors or the lower immune response in children [13]. A latest research suggested that SARS-CoV-2 invade cells through the angiotensin-converting enzyme 2 (ACE2) protein [14]. The ACE2 protein is affected by developmental factors, and it is possible that the ACE2 protein in children has lower binding affinity to the SARS-CoV-2 virus, or that the intracellular responses induced by ACE2 in alveolar epithelial cells are milder in children, when compared to adults. For adults diagnosed with COVID-19, many presented with a significant or progressive decrease in the number of peripheral blood lymphocytes, especially T lymphocytes [15]. However, the present case presented no significant depletion of lymphocytes, as the absolute number of lymphocytes were within the normal range. This was also observed in the other case reports on children with COVID-19 [3–5]. The depletion of lymphocytes were considered to be caused by a cytokine storm in the early stages of the disease, which stimulates the immune response, thereby consuming lymphocytes and suppressing cellular immune function [15]. The milder symptom observed in children might be due to the lower immune response. However, the exact mechanism requires further studies.

There are presently no specific antiviral treatment options proven to be effective for COVID-19. Interferon-α has been used in clinic for the treatment of viral pneumonia, acute upper respiratory infections, hand-foot-mouth disease, SARS and other viral infections in children. Clinical experience has shown that Interferon-α can reduce the viral load in the early stages of viral respiratory infection, reduce symptoms, and shorten the course of disease [16]. The present patient received interferon-α2b nebulization for 7 days, and the drug was discontinued after the disappearance of symptoms. With the lack of a control group, it was not possible to determine the exact effect of the treatment. However, no obvious adverse reactions were noted with the treatment regime.

In summary, the present pediatric case of COVID-19 was acquired through household transmission, and the symptoms were mild. The lymphocyte counts did not significantly decrease. The RNA of SARS-CoV-2 in stool and nasopharyngeal swabs remained positive for an extended period of time after the disappearance of symptoms. This suggests that attention should be given to the potential contagiousness of pediatric COVID-19 cases after clinical recovery. Further research is needed to understand the mechanism behind the limited lymphocyte response and milder symptomatology in children.

## Data Availability

All the data supporting our findings are contained within this article.

## Abbreviations

ACE2: Angiotensin-converting enzyme 2
COVID-19: Coronavirus Disease 2019
CT: Computed tomography
JAMA: Journal of American Medical Association
MERS: Middle East respiratory syndrome
PCT: Procalcitonin
RT-PCR: Reverse transcription polymerase chain reaction
SARS: Severe acute respiratory syndrome
SARS-CoV-2: Severe acute respiratory syndrome coronavirus 2
SARS: Severe acute respiratory syndrome
WHO: World Health Organization
